# Effects of the Prolong Life With Nine Turn Method (Yan Nian Jiu Zhuan) Qigong on Brain Functional Changes in Patients With Chronic Fatigue Syndrome in Terms of Fatigue and Quality of Life

**DOI:** 10.3389/fneur.2022.866424

**Published:** 2022-07-13

**Authors:** Fangfang Xie, Chong Guan, Yuanjia Gu, Yanli You, Fei Yao

**Affiliations:** ^1^Shanghai Municipal Hospital of Traditional Chinese Medicine, Shanghai University of Traditional Chinese Medicine, Shanghai, China; ^2^School of Acupuncture and Massage, Shanghai University of Traditional Chinese Medicine, Shanghai, China; ^3^Department of Traditional Chinese Medicine, ChangHai Hospital, Naval Medical University, Shanghai, China

**Keywords:** chronic fatigue syndrome, prolong life with nine turn method Qigong, fatigue, quality of life, ALFF, FC, fMRI

## Abstract

**Background:**

Chronic fatigue syndrome (CFS) is characterized by persistent fatigue, which often leads to physical and psychological damage. The Prolong Life with Nine Turn method (PLWNT) Qigong is considered as one of the complementary treatments for improving symptoms in patients with CFS. In this study, we used functional magnetic resonance imaging (fMRI) to explore the effects of PLWNT intervention on the subjects with CFS.

**Methods:**

Thirty four CFS patients were randomly divided into PLWNT group and cognitive behavioral therapy (CBT) group. Both groups were taught by a highly qualified professor at the Shanghai University of Traditional Chinese Medicine once a week and were supervised online during the remaining 6 days at home, over 12 consecutive weeks. We calculated the regional rs-fMRI index amplitude of low-frequency fluctuations (ALFF) for all subjects. To study the changes of the brain network, we used the brain regions with significant differences in ALFF as the regions of interest for whole-brain functional connectivity (FC) analysis. The Multi-dimensional Fatigue Inventory 20 (MFI-20) and Short Form 36-item Health Survey (SF-36) were used for clinical symptom assessment to explore the possible correlation between the rs-fMRI indicators and clinical variations.

**Results:**

The ALFF values of the right superior frontal gyrus (SFG), and left median cingulate gyrus (DCG) were increased, whereas those of the left middle occipital gyrus (OG), right middle OG and left middle temporal gyrus (MTG) were decreased in PLWNT group. The FC values between the DCG and middle temporal gyrus (MTG), and those between the left OG and the right OG were enhanced. In addition, the SF-36 were positively with the left OG (*r* = 0.524), SFG (*r* = 0.517), and DCG (*r* = 0.533), MFI-20 were negatively with the SFG (*r* = −0.542) and DCG (*r* = −0.578). These results were all corrected by FWE (voxel level *p* < 0.001, cluster level *p* < 0.05).

**Conclusion:**

CFS patients have abnormal regional spontaneous neuronal activity and abnormal functional connections between regions after PLWNT intervention. PLWNT can relieve the fatigue symptoms of CFS patients and improve their quality of life. The study was registered in the American Clinical Trial Registry (12/04/2018). Registration Number is NCT03496961.

## Introduction

Chronic fatigue syndrome (CFS), also be called myalgic encephalomyelitis, is a complex multisystem disease. It is commonly characterized by chronic fatigue lasting more than 6 months that is not alleviated after resting, and accompanied by cognitive dysfunction, sleep problems, autonomic dysfunction, post-exertional malaise, severely impaired activities of daily living, and/or pain in muscles or joints (1, 2). CFS is associated with poor health-related quality of life, even worse than cancer, multiple sclerosis, and stroke (3). In fact, ~25–29% of CFS patients are house- or bed-bound (4); over half of the patients are unemployed (5), and only 19% work full time (6). Although the mechanism underlying neurological dysfunction in CFS is not yet clear, CFS may be considered a prototypical disorder of brain connectivity (7, 8). The prevailing neuroimaging studies have suggested that the brain responds differently to a cognitive challenge in patients with CFS, with recruitment of wider regions to compensate for lower or higher information capacity (9). There is increasing neuroimaging evidence of functional and structural abnormalities in the brain of CFS patients, which suggested that the central nervous system is involved in this disorder and that at least some CFS patients may have an underlying neurological basis for their illness (10). Various drugs, such as non-steroidal anti-inflammatory drugs (NSAIDS), antidepressants, and COX-2 inhibitors, have been used to relieve and manage the symptoms (11, 12). However, the use of antidepressants is controversial and has significant side effects. Complementary and alternative medicine (CAM) is very popular among patients with diseases/illnesses of unknown etiology (13, 14). Cognitive behavioral therapy (CBT) seems to be a promising CAM for CFS (15–17), which address the fear of activity, maladaptive disease beliefs and symptom focusing by combining practical activities (18). However, persistent or sustained significant outcomes have been shown in few CFS patients (19). Many other CAM modalities, such as traditional Japanese herbal medicine (Kampo) (20), acupuncture (21, 22), and Qigong (23, 24) have demonstrated to be effective treatments and prevention methods in relieving fatigue, depression and insomnia.

Qigong (pronounced “chee gung”) is a therapeutic Chinese practice which has been used for thousands of years to optimize and restore energy (Qi) to the body, mind, and spirit (25); based on Taoist philosophy and Chinese medicine theory (26), it promotes health and vitality through gentle exercises for the breath, body and mind (27). Prolong Life with Nine Turn Method (PLWNT) is a type of Qigong that includes eight kinds of massage manipulations on the abdomen and a kind of upper body shaking. The exercise process focuses on practicing muscles, bones and skin externally and training the spirit, breath and energy internally, so that the essence is sufficient, and the internal and external coordination is unique. It is a unique method of prolonging life (28). Other forms of Qigong have been found to be equally temporarily effective (29, 30). The findings of PLWNT Qigong interventions may open a new page for the study of the effects of Qigong on humans, which can provide overall coordination to help the body achieve a state of relative equilibrium of yin and yang, dredging meridians, and restoring physiological function. Some studies have reported on the effects of massage techniques on fatigue and quality of life (31–33). The abdominal massage techniques included in PLWNT act on the movement of the pelvic and abdominal muscles, coordinated with diaphragmatic breathing. They may trigger contraction of the intestinal and rectal muscles, which can train the function of the intestines (34), but they also have an impact on the nervous system, including reductions in the excitability of the sympathetic nerve and enhancement of the excitability of the parasympathetic nerve to reduce fatigue when rubbing the internal organs (35). Other studies have shown that abdominal massage manipulation therapy of PLWNT can relieve muscle tension and regulate mood through electromyographic signals, neuromuscular synthesis, and nerve rhythm, thereby significantly relieving sleep disorders, fatigue, and depression symptoms of CFS patients and improving their quality of life (36). Our recently published protocol for this project predicts that PLWNT Qigong exercise can improve fatigue, sleep and depression in CFS patients, and improve the quality of life (28). However, the exact mechanism behind this phenomenon is still unknown.

Functional magnetic resonance imaging (fMRI) helps in non-invasive examination, localization, as well as lateralization of brain functions such as language and memory (37). It is a powerful imaging technique which has received substantial attention in brain disorder research (38–40). Recently, there has been an apparent shift in the focus of neuroscience research to studies dealing with the brain at “resting state,” which involve measurement of ongoing spontaneous brain activity amplitude of low-frequency fluctuations (ALFF) and mapping of interregional functional connectivity (FC) (41–43). Related studies have shown that the destruction of normal brain function may be the basis of the core symptoms of CFS, including fatigue, pain, anxiety, and depression, all of which affect the quality of life (44, 45). More than 80% of CFS patients report symptoms of anxiety and depression, especially sleep problems (46). Some neuroimaging studies have shown that CFS patients have reduced gray matter volume and white matter changes (47) in seed brain areas (regions of interest, ROI) related to CFS symptoms, such as memory (parahippocampal gyrus, PHG), motor skills (globus pallidus), emotional processing (anterior cingulate cortex), and higher-order neurocognitive functions, which have been widely used to assess the internal state of brain function (48). Compared with control group, CFS patients show more extensive activation of brain regions, manifested by functional abnormalities in the prefrontal, parietal, and limbic areas (49). However, few studies have investigated the effects of PLWNT intervention on CFS patients in terms of fatigue and quality of life.

In this study, we used fMRI to examine the effect of PLWNT intervention on fatigue and quality of life of CFS patients. The neuronal activity and the distribution of high-level node connections play a vital role in the transmission of information throughout the brain (50). Based on functional correlation and physiological basis, the entire neurological system of patients with CFS is usually affected (51, 52). A number of studies have shown that FC and ALFF indicators, which can detect central connections through voxel-based graphical analysis methods, are closely related to physiological indicators such as regional cerebral blood flow, oxygen, and glucose metabolism (53, 54). Therefore, we used the fMRI fast Fourier transform (FFT) algorithm to identify the changes in the central brain activity in the power spectrum observation area and to evaluate the synergy and antagonism of the BOLD signal at the voxel level to explore the effect of PLWNT on CFS. The purpose of this study was to investigate the neurological abnormalities in CFS patients at resting state and the regulatory effect of PLWNT on the functional network of CFS neuronal activity and functional connections.

## Materials and Methods

### Subjects

Thirty-four people were recruited for this study, 4 of them (2 in PLWNT group and 2 in control group) were excluded because their head motion was more than 2.5 mm translation (FD standard) during the scanning process. Thus, 30 subjects (15 in the PLWNT group and 15 in the control group) were finally included. The recruitment of the subjects was conducted from December 2018 to September 2019 at the Shanghai University of Traditional Chinese Medicine and Yueyang Hospital of Integrated Traditional Chinese and Western Medicine. We included hospitalized patients with a preliminary diagnosis of CFS, according to the latest Revise Guidelines for Treatment of Chronic Fatigue Syndrome in 2021 (55). The inclusion criteria were as follows: (1) age between 20 and 60 years; no gender requirement; (2) severe chronic fatigue that is unexplained after clinical evaluation and has a history of no <6 months; fatigue was not caused by the work performed during the trial, and the fatigue was not alleviated after rest; and (3) at least four of these eight symptoms (memory or concentration decline, failure to regain energy after sleep, sore throat, headache, lymph node tenderness, muscle pain, multiple joint pain, and myalgia after exertion for more than 24 h). The exclusion criteria were as follows: severe cardiovascular and cerebrovascular diseases, endocrine system diseases, motor system diseases, autoimmune diseases, infectious diseases, use of medications which may affect the judgment of the results. The detailed fundamental information of CFS subjects is available in our previously published protocol (28).

### Design

This study was designed as a randomized, parallel-controlled trial. The participants were randomly assigned to the PLWNT group and the control group. Qigong or cognitive behavior education and learning were conducted at Shanghai University of Traditional Chinese Medicine, each taught by a senior exercise teacher and a psychology teacher. Exercise was practiced at home no <6 times a week. The total duration of the study was 12 weeks. We distributed exercises/learning notebooks every week for recording their exercises/learning until the end of the experiment. Detailed information is available in the previously published protocol (28). The study was conducted in accordance with the Declaration of Helsinki and the International Code of Ethics for Biomedical Research Involving Human Subjects. It was approved by the Ethics Committee of Yueyang Hospital of Integrated Traditional Chinese and Western Medicine (Ethics Approval Number: 2018-043), and registered in the American Clinical Trial Registry (12/04/2018), Registration Number is NCT03496961.

### Randomization and Concealed Allocation

CFS patients who meet the criteria and sign the consent form will be randomly divided into PLWNT group and CBT group with a ratio of 1:1. The specific randomization procedure is as follows. To avoid possible selective bias, a statistician who did not participate in the trial process used a computer software program (Strategic Applications software, version 9.1.3; SAS Institute Inc., Cary, NC, USA) to generate a random number table sent to a specially designated administrative staff at the Test Management Center of Shanghai University of Traditional Chinese Medicine to ensure safety. They didn't participate in trial recruitment or treatment of participants. The administrative staff would store each patient's identity information, treatment method, time, and location in an opaque envelope based on random numbers. After that, they handed the sealed envelope to the research team, and the research team would print and save it in the original form.

### Intervention

#### PLWNT Group

**Figure 1:**
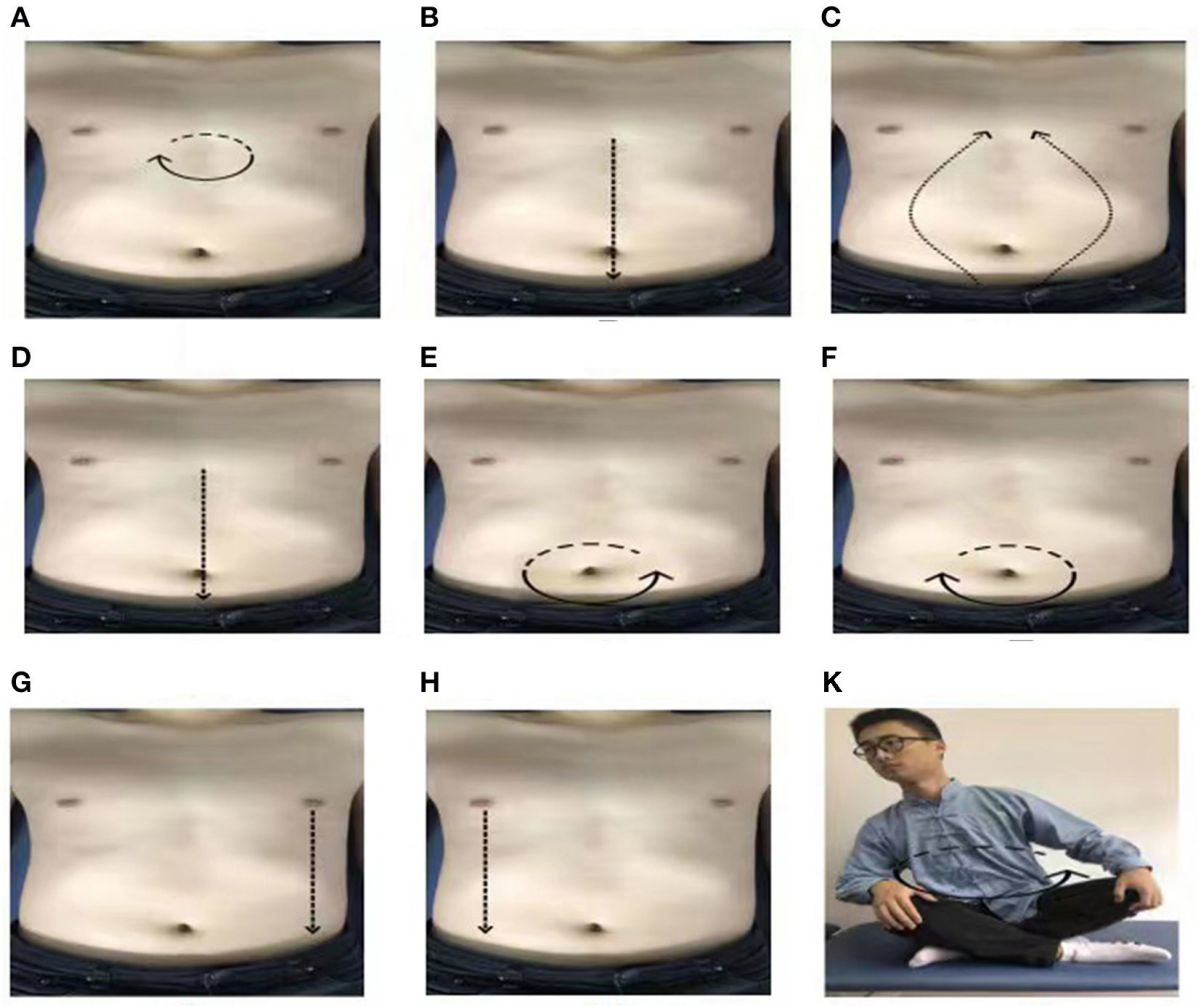
The postures of PLWNT. **(A)** Press and knead acupoint in Danzhong. **(B)** Rubbing from Danzhong Acupoint to Pubic Symphysis. **(C)** Rubbing from Pubic Symphysis to Danzhong Acupoint. **(D)** Pushing from Danzhong Acupoint to Pubic Symphysis. **(E)** The right hand massages the abdomen by the left circle. **(F)** The left hand massages the abdomen by the right circle. **(G)** Pushing with the right hand from the left breast to the groin. **(H)** Pushing with the left hand from the right breast to the groin. **(K)** Turn left and right. Every movements will be carried out 21 times. PLWNT, prolong life with nine turn method.

Qigong professors at Shanghai University of Traditional Chinese Medicine, who have been engaged in Qigong education for at least 5 years, were in charge for the supervision of the exercise and corrected the exercise posture during the entire intervention period for 1 h every Sunday. Professional Qigong teachers spent 10 min to perform stretching and relaxation exercises, as well as movement introductions and demonstrations. In addition, they explained precautions during the process and answered the participants' questions. Subsequently, the teachers gave 30 min of action guidance and correction to each of the participants. Finally, all of the participants practiced Qigong for 20 min together. For the remaining 6 days of the week, all the participants in the WeChat cluster had to join WeChat video supervision and practice for 30 min at 6 o'clock every day. If some of the participants found it inconvenient, the private WeChat video surveillance exercise was conducted. Each participant filled in the “Working Practice Record” after every exercise. The entire practice process lasted for 12 weeks. The average amount of abdominal stimulation for the first eight rubbings was 0.5 ± 0.1 kg. Before we do the exercises, we let patients wear manual stimulation data gloves, and monitor the strength of the manual in real time in the LABVIEW2017 software, so that patients can feel the amount of stimulation. The content of PLWNT Qigong intervention was the same as in our previous research (28). The nine specific forms of manipulations are shown in Figure 1. This picture comes from our previously published protocol (28).

##### Step 1. Preparatory Position

During this step, the participant should relax their whole body, concentrate their thoughts, breathe evenly, place their tongue against the upper jaw, hold their Dantian with their mind, and progress through the exercise step by step.

##### Step 2. PLWNT's First Eight Types of Abdominal Massage

Press the Danzhong acupoint (under the xiphoid process) with the middle three fingers in both hands and make a circle 21 times from the left, within 3 min.

With three fingers of both hands, rub down from the Danzhong acupoint and move to the pubic symphysis below the umbilicus. Repeat 21 times within 3 min.

With three fingers in both hands, rub up from the pubic symphysis from two sides back to the Danzhong acupoint until the hands are overlapped. Repeat this 21 times within 3 min.

With three fingers of both hands, push down from the Danzhong acupoint and push it straight to the pubic symphysis. Repeat 21 times within 3 min.

Rub the abdomen with the right hand from the left 21 times within 3 min.

Rub the abdomen with the left hand from the right 21 times within 3 min.

Place the left hand on the left side of the lower waist and kidney, with the thumb forward, and, using the four fingers supporting the back, gently pinch it; meanwhile, with three fingers of the right hand, push straight from the bottom of the left breast to the groin, and repeat this 21 times in 3 min.

Place the right hand on the right side of the lower waist and kidney, with the thumb forward, and, using the four fingers supporting the back, gently pinch it; additionally, with three fingers on the left hand, push straight from under the right breast to the groin, and repeat this 21 times in 3 min.

##### Step 3. Seated Rocking Method

Sit cross-legged, the participant should hold their hands up slightly and press them on the knees. The toes of both feet should be slightly bent. The participant should revolve the upper body clockwise 21 times and then counterclockwise 21 times.

#### Control Group

CBT therapists with sufficient professional qualifications [e.g., diploma in CBT, or other professionally accredited qualifications involving CBT as a major part of training (e.g., a clinical or counseling psychologist degree)] were invited to conduct CBT by giving lectures or psychological consultations on the prevention and treatment of CFS for 1 h, once a week. For the remaining 6 days of the week, all the participants in the WeChat cluster had to join WeChat video surveillance and listen to lectures for 30 min every day. If some of the participants found it inconvenient, the private WeChat video surveillance Microsoft PowerPoint learning was conducted. Each participant was asked to fill in the “Working Practice Record” after each video study to ensure that the other conditions were the same as those of the PLWNT group. The entire practice process lasted for 12 weeks. The detailed information is available in the previously published protocol (28).

### Data Acquisition

The fMRI data were obtained from all the participants, using the 3.0-T Trio Siemens System at Yueyang Hospital of Integrated Traditional Chinese and Western Medicine, Shanghai University of Traditional Chinese Medicine, Shanghai, China. The 32 head coils were used for scanning. In resting state BOLD signal acquisition single excitation gradient echo-plane imaging (GE-EPI) sequence, 30 participants were scanned as follows: repetition time (TR) = 1,900 ms; effective echo times (TE) = 2.93 ms; sagittal slices = 188; thickness/skip = 1.2/0.6 mm; field of view (FOV) = 256 × 256 mm^2^; matrix = 240 × 256 mm^2^; voxel size = 1.0 × 1.0 × 1.0; phase encoding direction = A > > P; and flip angle (FA) = 90°. The subjects were asked to close their eyes and rest for 10 min, and not to think about anything before the scan. They were instructed not to move their head during data collection. We obtained 242 three-dimensional image volume parameters as follows: TR = 2,000 ms; TE = 30 ms; section thickness = 1 mm; sagittal slices = 32; FOV = 256 × 256 mm^2^; matrix = 64 × 64 mm^2^; and FA = 90°. Both groups of the subjects were tested before and after the experiment.

The structural imaging data were processed by using MATLAB 2015a (MathWorks, Natick, MA, USA) and SPM12 (Wellcome Department of Cognitive Neurology, UK). MRICON was used to convert DICOM scan format to NIFTI format and eliminate the 10 abnormal time points before the scan. Next, we eliminated the difference in the scanning time of the fMRI signal compartment and the artifacts caused by slight head movement. We excluded the subjects whose head motion was >2.5 mm translation (FD standard). Then, we matched the functional image after head movement correction with the cut structure image to achieve the mapping of the individual image to the standard brain template space, and then unified it to the Montreal Neurological Institute (MNI) space and re-sampled at 3 × 3 × 3 mm voxel size. Subsequently, we co-registered the functional image with the T1 image, used 10 mm full-width half-maximum (FWHM) to check the space for smoothing, de-trended the resampled image, and then calculated the ALFF of each participant index. To study the changes of the brain network, we used the brain areas with significant differences as the regions of interest for the whole-brain FC analysis.

### Primary Index

#### Amplitude of Low-Frequency Fluctuations

The ALFF uses the level of the BOLD signal and the FFT algorithm to convert the smooth signal of each voxel from the time domain to the frequency domain, thereby obtaining changes in brain activity in the power spectrum observation area. ALFF mainly calculates the value distributed in the range of 0.01–0.08 Hz after the square of the power spectrum, reflecting the strength of neuronal activity in each brain area.

### Secondary Index

#### Functional Connectivity

FC is the degree of correlation of BOLD sequences in different brain regions in the time dimension. Each voxel of the brain area contains a time series, which represents the level of the area-dependent signal changes over time. We used brain regions with significant differences in ALFF as regions of interest to perform whole-brain FC analysis to study the changes in the brain network. The changes in the time series can determine that the brain regions that positively correlate with the BOLD signal are functionally synergistic and negative. The related brain areas are antagonistic. The most common FC is voxel-wise FC, that is, the functional connection based on seed points. The specific process is to select a seed point and calculate the correlation between the seed point and the BOLD signal of all voxels in the whole brain.

#### MFI-20

The MFI-20 is widely used for CFS measurement of mental and physical fatigue (56), including a total of 20 items, including five dimensions of general fatigue, physical fatigue, mental fatigue, reduced activity, and reduced motivation. Each item can be scored on a scale of zero to five points, and the total possible score is 100 points. The higher the score is, the more severe the fatigue is. The MFI-20 was found to have good internal consistency (Cronbach's alpha = 0.89) and reliability (Pearson correlation of the total score = 0.73) (57).

#### SF-36

The health status will be assessed by using the SF-36, which includes 36 questions related to an individual's quality of life that is summarized in two component summary scores: the physical component summary and the mental component summary scores. The SF-36 evaluates the following eight physical and mental health areas: physical functioning (PF), physical role functioning (RP), bodily pain (BP), general health (GH), vitality (VT), social role functioning (SF), emotional role functioning (RE), and mental health (MH). Each of the eight areas is scored on a scale of 0–100, where a higher score indicates better health subjectively. These scores are calculated from the questionnaires that were described previously (58). Cronbach's alpha for SF-36 is between 0.84 and 0.88 (59). It also has good construct validity and content validity (60).

### Statistical Analysis

The clinical data were analyzed using SPSS 21.0 software package (SPSS version 21.0, SPSS Inc. Chicago, IL, USA). For measurement data, such as age and scale score, the average value ± standard deviation (S) was used. The measurement data complying with the normal distribution and the test of homogeneity of variance were tested by an independent sample *t*-test. For non-normally distributed measurement data, pairwise comparisons between groups were based on the Mann–Whitney non-parametric test for two independent samples. In addition, to evaluate the abnormal brain activity of CFS patients, after data pre-processing, we used SPM12 to perform a two-sample *t*-test between the ALFF average image groups, with *p* < 0.05 and the brain area corrected by FWE (family-wise error) considered statistically significant at *p* < 0.05. Then, we took the ALFF different brain areas as the points of interest and counted the differences in FC between the ROI and the whole brain. The specific process was to make functional connections between the seed areas (ALFF different brain areas) and all voxels of the whole brain. We used Fisher transform to obtain the correlation value of the normal distribution z scores of the two sets of images with $\text{z }=\;{\text{log}}_{e}\frac{1+r}{1-r}$ (r is the correlation coefficient), which obtained the FC average image. A two-sample *t*-test was performed between the groups. Differences were statistically significant at *p* < 0.05 and the brain area corrected by FWE. We used XJVIEW to present the results. The Spearman correlation analysis was used to study the possible relationship between the ALFF brain nerve activation area and the clinical features of the MFI-20 and SF-36 scale scores.

## Results

### Demographic and Clinical Characteristics

**Figure 2:**
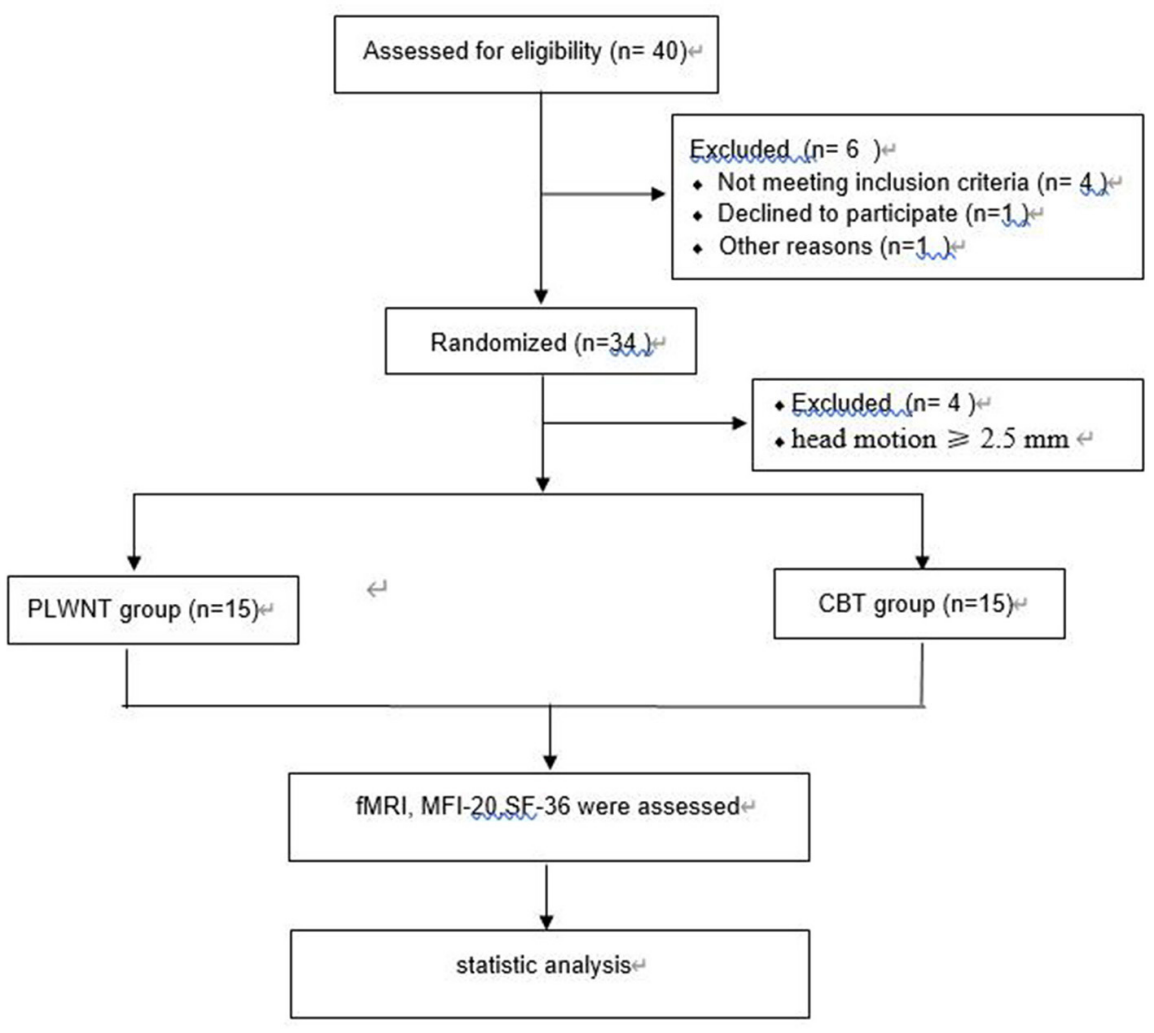
Flow diagram of the study design.

Thirty-four people were recruited for this study, 4 of them (2 in PLWNT group and 2 in control group) were excluded because their head motion was more than 2.5 mm translation (FD standard) during the scanning process. Finally, 15 in the PLWNT group and 15 in the control group were finished tested using fMRI in a randomized controlled trial before and after the study. Flow diagram of the study design is shown in Figure 2. The clinical and demographic characteristics and the intergroup differences are shown in Table 1. There were no significant differences between PLWNT group and control groups in terms of age, weight, height, gender and education (*p* > 0.05), which confirmed the two groups were comparable.

**Table 1:** Demographic and clinical characteristics of the patients.

	**PLWNT (*n* = 15)**	**Control (*n* = 15)**	***P*** **-value**
Age (year)	37.943 ± 11.3447	37.343 ± 9.864	0.935
Weight (kg)	59.804 ± 10.893	61.943 ± 12.061	0.557
Height (cm)	163.514 ± 6.679	165.000 ± 7.376	0.209
Gender (M:F)	7:8	6:9	0.500
Education (year)	11.823 ± 3.25	11.232 ± 2.86	0.641
MFI-20	9.53 ± 4.051	10.27 ± 2.685	0.564
SF-36	39.67 ± 11.568	44.33 ± 10.328	0.254
**Inclusion criteria**
Meet 4 out of 8	5	6	/
Meet 5 out of 8	5	4	/
Meet 6 out of 8	3	4	/
Meet 7 out of 8	1	1	/
Meet 8 options	1	0	/

### Primary Index

#### ALFF Changes

**Figure 3:**
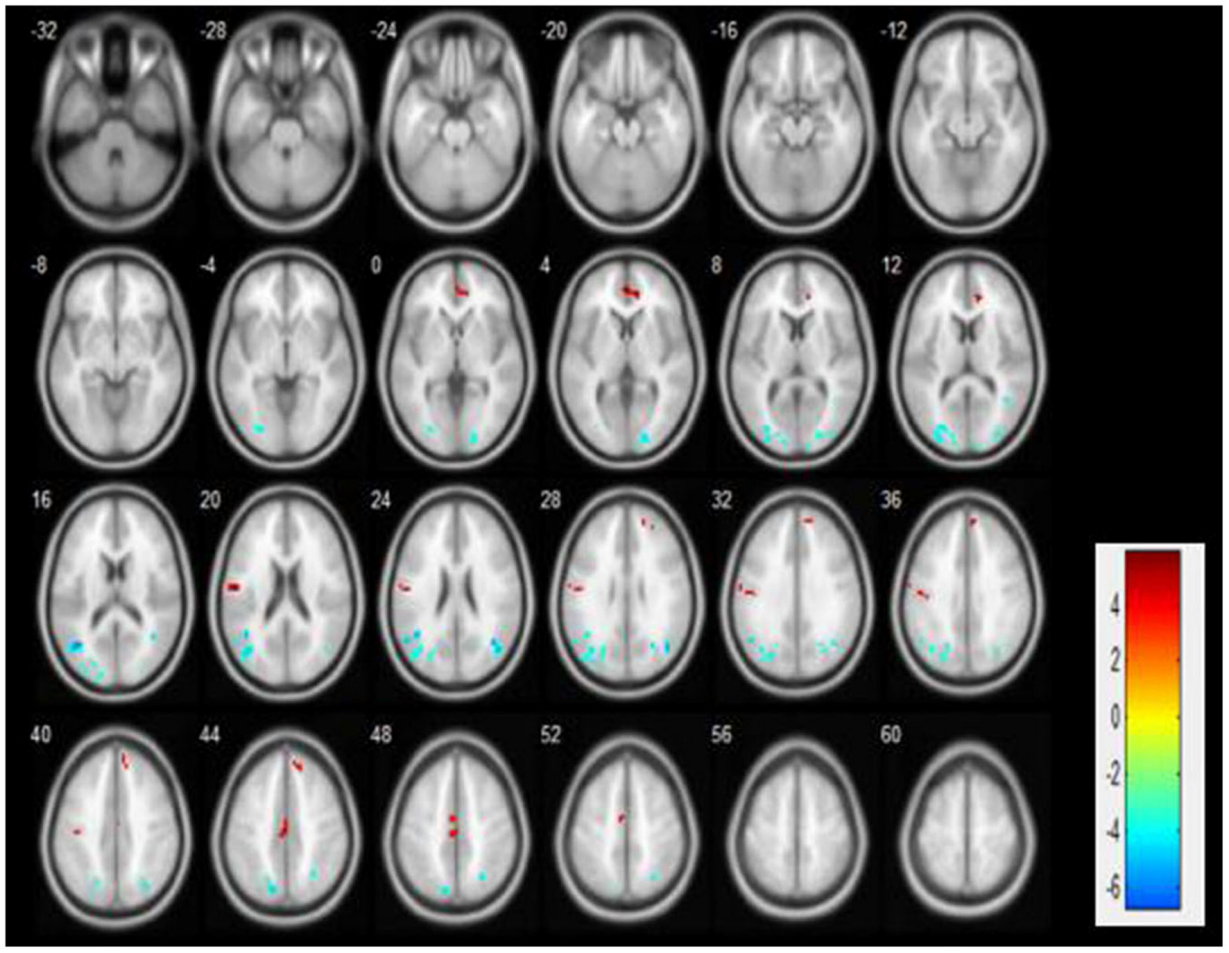
Compared with the education group, the ALFF brain area changes in the PLWNT group. Red shows enhanced area, green shows reduced area, based on FWE correction, with voxel level *p* < 0.001 and cluster level *p* < 0.05.

We observed significant activation of ALFF neuronal activity in CFS patients (*p* < 0.05). Compared with the control group, the brain areas with significantly enhanced ALFF value in the PLWNT group were the right superior frontal gyrus (SFG) and left median cingulate gyrus (DCG). In contrast, the areas with decreased ALFF value in the PLWNT group included the left middle occipital gyrus (OG) and left middle temporal gyrus (MTG). These regions were all corrected by FWE with voxel level *p* < 0.001 and cluster level *p* < 0.05 (Table 2, Figure 3).

**Table 2:** Compared with the control group, the brain areas enhanced and reduced ALFF value in the PLWNT group.

**Cluster**	**L/R**	**Regions**	**X**	**Y**	**Z**	**Voxel**	***T*** **-values**
Cluster 1	L	OG	−24	−81	12	78	−4.7812
Cluster 2	R	OG	21	−90	6	55	−5.0478
Cluster 3	R	SFG	6	51	36	35	4.6676
Cluster 4	L	DCG	0	−18	45	35	5.0678
Cluster 5	L	MTG	−39	−63	15	197	−6.6237

*These regions are all corrected by FWE, with voxel level p < 0.001 and cluster level p < 0.05*.

### Secondary Index

#### FC Changes

**Figure 4:**
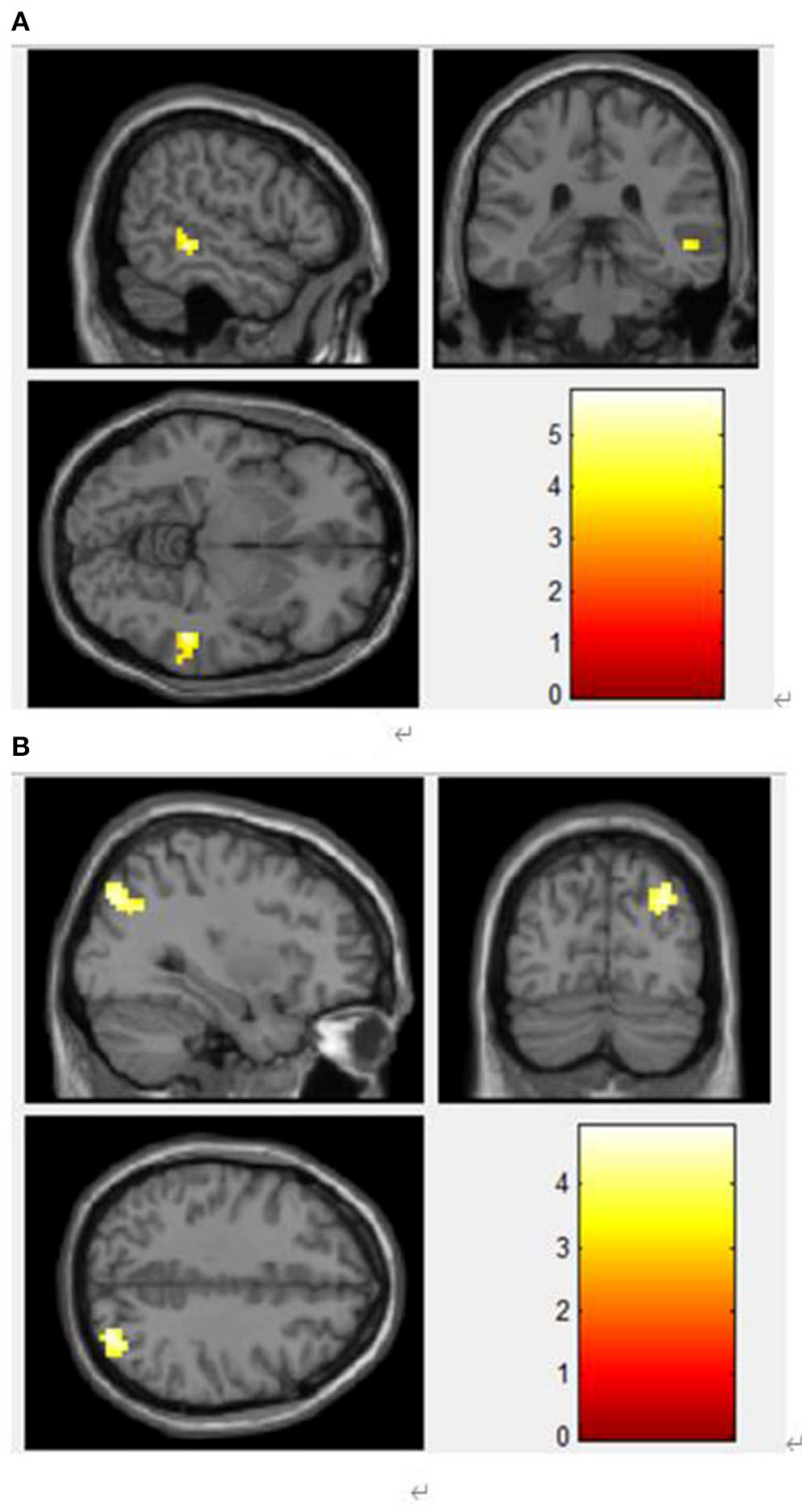
**(A)** Functional connection enhancement area of DCG and MTG. **(B)** Functional connection enhancement area of the left OG and the right middle occipital gyrus.

To study the changes in the brain network, we took the brain regions with significant differences in ALFF as the regions of interest (ROI) for whole-brain FC analysis, and finally obtained two ROIs: OG and DCG. Compared with the control group, the FC values between the DCG and MTG (Table 3, Figure 4), and those between the left OG and the right OG were enhanced (Table 3, Figure 4).

**Table 3:** Compared with the control group, the FC values between the DCG and MTG.

**ROI**	**Regions**	**X**	**Y**	**Z**	**Voxel**	***T*** **-value**	***P*** **-value**
ROI-1 DCG	MTG	51	−39	−6	47	5.83	*P* <0.05
ROI-2 OG	Right middle occipital gyrus	30	−81	39	76	4.92	*P* <0.05

#### Clinical Symptom Scale

In our study, we showed the results of these 30 individuals with Multi-dimensional Fatigue Inventory 20 (MFI-20) and Short Form 36-item Health Survey (SF-36). The results showed that the change of MFI-20 after PLWNT intervention was 9.53 ± 4.051 VS. 4.80 ± 2.111 (*P* < 0.05), the change of SF-36 was 39.67 ± 11.568 VS. 69.67 ± 15.407 (*P* < 0.05; Table 4).

**Table 4:** The results of 30 CFS patients with MFI-20 and SF-36.

	**PLWNT group (15)**			**CBT group (15)**			**Between group *P***
	**Before**	**After**	**Within-Group *P***	**Before**	**After**	**Within-Group *P***	
MFI-20	9.53 ± 4.051	4.80 ± 2.111	0.000*	10.27 ± 2.685	7.13 ± 3.292	0.002*	0.202
SF-36	39.67 ± 11.568	69.67 ± 15.407	0.000*	44.33 ± 10.328	59.67 ± 13.425	0.001*	0.016

*P < 0.05.

### Correlation Between the fMRI Parameters and the Clinical Features

**Figure 5:**
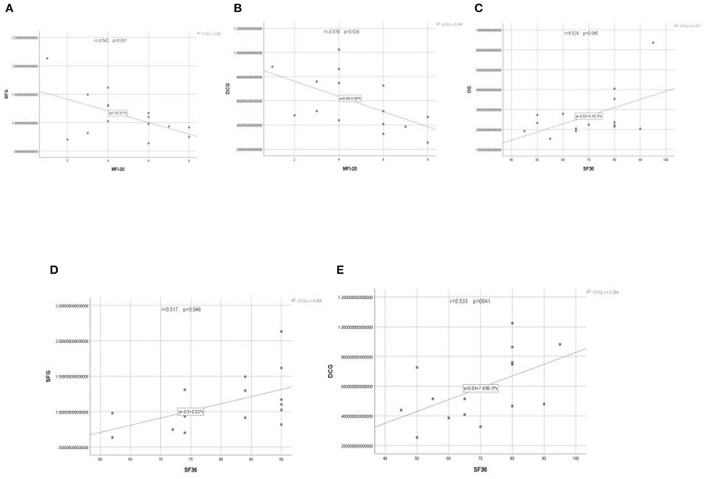
**(A–E)** The relationship between the changes of neuronal activity (ALFF) in OG, SFG, and DCG brain regions and the scores of patients on the MFI-20 and SF-36 scales.

Since fatigue and quality of life were the focus of the study, the additional correlation analysis between the scales and brain activation areas outcomes were performed. The Pearson correlation coefficient was used to test the relationship between the scales of score of the Multidimensional Fatigue Inventory (MFI-20), Health Survey Short Form (SF-36) and the brain activation of OG, SFG, and DCG. PLWNT interfered with neuronal activity and had a significant impact on the scores of SF-36 and MFI-20. The clinical symptoms in SF-36 were positively with the left OG (*r* = 0.524), SFG (*r* = 0.517), and DCG (*r* = 0.533), and clinical symptoms in MFI-20 were negatively with the SFG (*r* = −0.542) and DCG (*r* = −0.578). *P*-values were all < 0.05. The results of the correlation analysis are shown in Figures 5A–E.

### Adverse Events

Table 5 displays data from a total of 2 participants who reported adverse events in our study. However, they were all defined as possibly not or definitely not related to the exercises. One of the cases was clearly not related to the qigong exercises. It was caused by low blood sugar caused by fasting, which was relieved after eating breakfast. The other case was due to the patient's past history of hands and improper abdominal massage, which resulted in milder symptoms and resolved after 3days.

**Table 5:** Adverse event.

**Symptom**	**Patients numbers**	**Start date**	**End date**	**Relationship**	**Treatment**	**Action related to intervention**	**Outcome**
Dizziness	1	20190512	20190512	Probably not	Measure blood pressure and eat breakfast	No change	Cured
Left thumb pain	1	20190621	20190624	Definitely not	None	No change	Cured

## Discussion

The degree of fatigue of CFS patients is closely related to life quality, due to its potential social impacts, as well as its bad influences on body and mind. PLWNT Qigong is an treatment of traditional Chinese medicine, which is a physical and mental exercise that adjusts body, mind and spirit simultaneously. As one of traditional physical exercises, PLWNT includes eight kinds of massage manipulations on the abdomen and one of upper body shaking, which focus on abdomen acupoints stimulation to organically strengthen the body health and improve breathing and spirit. At the same time, it uses the heat of palm friction to promote the circulation of Qi and blood throughout the body. Thus, it is an optimal method of improving fatigue, body health and further life quality. In addition, according to the theory of traditional Chinese medicine, the abdomen is where the spleen and the stomach are located, which governs the muscles and is closely related to CFS (61). During the practices of PLWNT, posture training of the abdomen and limbs with mental concentration improves the static strength of the muscles, self-control and coordination ability of the autonomic nervous system, as well as increase the amount of blood circulating to the heart and brain to relieve fatigue and improve life quality (62, 63). The previous study suggested that PLWNT can effectively relieve the clinical fatigue symptoms of CFS patients to improve life quality (64).

This study used resting-state fMRI to analyze the effect of PLWNT intervention on the activation of brain network neurons and FC changes in CFS patients. The change in resting state network (RSN) is related to the lack of mental activity, which seriously affects the quality of life (65). At present, RSN is considered to have strong spontaneous activity. It is also the most common neural network for evaluating quality of life, involving cognitive control, attention, language processing, and working memory (66), including the OG, angular gyrus, MTG, SFG, and DCG (67). RSN brain area is related to the maintenance of the brain's alertness to the outside world and introspection (68). Previous studies have shown that CFS can lead to impaired RSN function, which is manifested when performing externally targeted tasks such as cognitive memory tasks (69, 70). As shown in our study, the OG and MTG of the PLWNT group of patients belong to RSN and have a lower ALFF value compared with the control group. It suggests that long-term fatigue, insomnia, and poor quality of life in CFS patients can cause damage to the brain's advanced cognitive memory function. The injury of a certain center does not permanently remove the function managed by the center, the function can be compensated by other areas to restore the function to a certain extent after exercise (71). This may be a neural compensation mechanism for the functionally damaged brain areas in CFS patients after PLWNT treatment. The ALFF values of the SFG and DCG were all increased. This shows that when CFS patients suffer from fatigue and sleep disturbance which affect their quality of life, there are new strengthened brain areas which continue to complete specific neuronal activities and brain functional activities.

Our study showed that 12 weeks of PLWNT intervention had a positive effect on ALFF and FC of abnormal brain regions of OG, SFG, and DCG in patients with CFS. The FC values between the DCG and MTG, and between the left OG and the right OG were all enhanced. In previous studies, these brain areas have been linked to fatigue and quality of life (72–74). We used correlation analysis to observe the relationship between ALFF, FC, and the improvement of clinical symptoms. The results of our study showed that among patients receiving Qigong, changes in the ALFF and FC values of DCG, SFG, and bilateral OG suggest that patients with CFS have increased hemodynamic response to local neural activity or the brain's compensatory response to fatigue, these brain activation areas positively correlated with the clinical symptoms in SF-36 and negatively correlated those in MFI-20 in terms of fatigue, physical pain and lack of energy, thereby suggesting that PLWNT may reflect the quality of life of CFS patients through the DCG, SFG, and OG neuronal activity objectively. Compared with other analyses, ALFF analysis can suppress non-specific signals more effectively, in order to significantly improving the neuron specificity for detecting spontaneous activity in brain regions (75). The FC analysis focuses on the similarity of spontaneous brain activity within and between regions, with the ALFF activation area as the point of interest (76). Changes in the ALFF and FC values of DCG, SFG, and bilateral OG suggest that patients with CFS have increased hemodynamic response to local neural activity or the brain's compensatory response to fatigue. These findings provide support that PLWNT Qigong intervention may actively improve the clinical symptoms of CFS patients. DCG, SFG, and OG can reflect the spontaneous neural activity of the brain and the activation of CFS abnormal brain areas. The changes in DCG, SFG, and OG can help to understand the changes in brain nerve function in CFS patients after exercises.

The defects of the somatic motor center seem to be related to the higher levels of fatigue in CFS, somatic pain, energy disorders and other aspects of the quality of life (77–79). Researchers have recognized the importance of the motor function of the somatic motor center in explaining mental fatigue early, but the structure of the somatic motor center and OG is not sufficient to explain the model. Subsequently, brain area networks including SFG, DCG, and MTG have also been shown to be related to fatigue (80, 81). SFG corresponds to the somatosensory center, which is mainly responsible for processing spatial information, attention control, and somatosensory information. SFG is of great significance to the adjustment of the quality of life such as fatigue, anxiety, and depression of CFS patients, as well as for the improvement of functional activities, learning, and memory (82–84). Recent studies have found that the SFG area is widely activated when CFS patients participate in activities, although it is not clear whether these activations are caused by positive or negative emotions, it shows that severely fatigued brains need to activate the right frontal lobe and adjacent areas (83). Compared with the control group, the SFG neuron activity in the PLWNT group was abnormally increased. Consistent with the results of our study, research has shown that the white matter of the brain area related to cognition promotes information transmission in the brain, which makes the nervous system fast and effective (85). Any disorder of these neurological functions will affect the quality of life, including memory, attention, energy, and executive function, as shown in CFS (85–87). In addition, SFG is related to deficits in working memory, impaired attention, poor motor coordination, and inability to concentrate vision. This area plays an important role in connecting the frontal and temporal lobes (88, 89). The above findings may reflect that PLWNT intervention increased the activation of SFG neurons and the functional integration with MTG. This change may improve higher-level processes such as fatigue and quality of life.

Higher-order level cognitive dysfunctions, such as those of memory and cognition, are well-known in CFS (90). The recent studies have also documented the effects of basic sensory processing deficits on quality of life (91, 92). In CFS patients, there are also perceptual defects of the visual system (91–93). In the human brain, the OG is the main brain region for visual processing, which is involved in memory acquisition (94). Bilateral OG cortex contains topographic maps of size and orientation preference, in which neural responses to stimulus sizes and stimulus orientations are modulated by intraregional lateral connections. We propose that these lateral connections may underlie the selective influence of PLWNT Qigong on visual perception (95, 96). Researchers have found that the white matter of the right suboccipital tract of Gulf War syndrome veterans with visual neglect was damaged in connection with the occipital cortex, by using diffusion-tensor imaging, which was manifested by severe fatigue, sleep, and decreased quality of life (97). In our study, the OG structure of CFS patients showed decreased neuronal activity. These convergent results emphasize the possibility that memory decline, unrecoverable fatigue, and the decreased quality of life in CFS patients may be related to OG dysfunction. Apart from the increase in OG neural activity, this study revealed that Qigong exercises actively increased FC between the bilateral OG. These effects may be related to the ability of Qigong to change the brain's functional networks related to the processing of external visual stimuli (83, 98). To our knowledge, the enhancement of FC in OG has not been reported to play a role in CFS quality of life, which may suggest that the FC of the OG dysfunction may affect the quality of life of CFS patients and may also be involved in the pathogenesis of CFS.

The fatigue symptoms of CFS and the decline in quality of life are closely related to the transition network that connects cognitive and emotional feelings (66, 99). There is numerous research evidence (100–102) that DCG participates in a series of functions; not only it can process emotions, feelings and attention, but it can also participate in the regulation of fatigue. The gray matter creatine phosphate of CFs patients is reduced, which indicates that CFS consumes more energy than normal patients. The enhancement of these activated abnormal brain regions and the enhancement of the functional connection of MTG may be related to the high-energy compensation mechanism. Beyond that, pain is also a common symptom of CFS and an important factor that affects the quality of life. These pain disorders can occur in multiple locations, from the cerebral cortex to the spinal cord, and is considered to be the damage to the central nervous system may cause the neurotransmitter involved in analgesia to be abnormal release (103). If DCG has obstacles in receiving and processing pain information, then the brain is more sensitive to pain (104). Previous studies have also confirmed that this was mainly related to the core activation of the anterior cingulate gyrus, SFG, occipitotemporal area, and DCG in our study, which was typical features of pain management (105). More importantly, Shan et al. (9) found that SFG and OG neuron activities in CFS patients positively correlated with SF-36 by comparing CFS patients with healthy people, which is consistent with our findings that the brain activation areas positively correlate with the clinical symptoms in SF-36. Based on these observations, we can infer that PLWNT can reduce the fatigue symptoms and improve the quality of life in CFS patients by activating related DCG-related brain regions.

Although our findings provide new and objective insights into the effects of PLWNT intervention on the brain function of CFS patients (including fatigue symptoms and quality of life), there are still some limitations that need to be further addressed. First of all, the selection criteria for CFS patients in this study were only based on self-rating scales; this may have resulted in irregular requirements for the inclusion of patients. However, we limited the age of participants to 20–60 years to reduce the likelihood of chronic fatigue symptoms and poor quality of life caused by diseases and age. Second, participants should be blinded ideally, but this is difficult to achieve in non-drug trials. However, we tried our best to ensure that laboratory technicians, data management personnel, and statisticians did not participate in recruitment and data processing, which to a certain extent ensured the authenticity of the data. Finally, our results indicating the brain regions with enhanced neuronal activity and functional connectivity in patients with CFS after PLWNT intervention need to be verified in a larger sample.

## Conclusions

This study showed that PLWNT can relieve the fatigue symptoms of CFS patients and improve their quality of life. We also found that CFS patients had abnormal regional spontaneous neuronal activity and abnormal functional connections between regions after PLWNT intervention. There were also changes in the activation of the brain regions, improving the quality of life related to fatigue symptoms and physical pain, which were linearly related to the clinical symptoms in MFI-20 and SF-36. These findings provide a new perspective for the role of traditional medical interventions such as Qigong in medicine, and may provide guidance for the diagnosis and prevention of CFS.

## Data Availability Statement

The original contributions presented in the study are included in the article/supplementary materials, further inquiries can be directed to the corresponding authors.

## Ethics Statement

The studies involving human participants were reviewed and approved by the Ethics Committee of Yueyang Hospital of Integrated Traditional Chinese and Western Medicine (Ethics Approval Number: 2018-043). The patients/participants provided their written informed consent to participate in this study. Written informed consent was obtained from the individual(s) for the publication of any potentially identifiable images or data included in this article.

## Author Contributions

FX performed the study. FY and YY contributed to data analysis. The manuscript draft was revised by CG and YG. The project was conceived and designed by all the authors. All authors contributed to the article and approved the submitted version.

## Funding

This work was supported by the National Natural Science Foundation of China under Grant No. 81774443 and 82105038 (to FY and FX). The funding sources were not involved in study recruitment, data processing, and publication of papers.

## Conflict of Interest

The authors declare that the research was conducted in the absence of any commercial or financial relationships that could be construed as a potential conflictof interest.

## Publisher's Note

All claims expressed in this article are solely those of the authors and do not necessarily represent those of their affiliated organizations, or those of the publisher, the editors and the reviewers. Any product that may be evaluated in this article, or claim that may be made by its manufacturer, is not guaranteed or endorsed by the publisher.
